# Comparison of the Amplitude of Accommodation Measured Using a New-Generation Closed-Field Autorefractor with Conventional Subjective Methods

**DOI:** 10.3390/diagnostics12030568

**Published:** 2022-02-23

**Authors:** Piotr Kanclerz, Karolina Pluta, Hamed Momeni-Moghaddam, Ramin Khoramnia

**Affiliations:** 1 Department of Ophthalmology, Hygeia Clinic, 80-286 Gdansk, Poland; karolinawieslawapluta@gmail.com; 2 Medical University of Gdansk, 80-210 Gdansk, Poland; 3 Health Promotion Research Center, Zahedan University of Medical Sciences, Zahedan 9816743463, Iran; hmomeni_opt@yahoo.com; 4 The David J. Apple International Laboratory for Ocular Pathology, Department of Ophthalmology, University of Heidelberg, 69120 Heidelberg, Germany; ramin.khoramnia@med.uni-heidelberg.de

**Keywords:** amplitude of accommodation, minus-lens method, push-up method, open-field autorefractometry

## Abstract

**Purpose:** This study aims to compare and assess the agreement of the objective amplitude of accommodation (AA) measured using a new-generation closed-field autorefractor with conventional subjective methods. **Methods:** In total, 84 healthy individuals with an age range of 19 to 50 years participated in this cross-sectional study. AA was measured objectively with a Nidek autorefractor (AR-1a; Nidek Co., Ltd., Tokyo, Japan) and subjectively using push-up (PU) and minus-lens (ML) methods in a random order. Comparison between different methods was performed using repeated-measures analysis of variance and the Bonferroni test for pairwise comparisons. In addition to the Pearson correlation, the Bland and Altman method and the intraclass correlation coefficient were used to determine the agreement between the three techniques. Only the right-eye results were used for analysis. **Results:** AA measured using the Nidek autorefractor (3.43 ± 1.94 D) was significantly lower than that measured with PU (7.67 ± 2.38 D; *p* < 0.001) and ML (7.60 ± 2.81 D; *p* < 0.001) methods. The difference between the subjective methods was not significant statistically (*p* = 1.0). The correlation for Nidek measurements and PU and ML methods was moderate (*r* = 0.5502 and *r* = 0.6832, respectively), while it was strong when comparing subjective methods (*r* = 0.7821). The limits of agreement for Nidek vs. PU, Nidek vs. ML, and PU vs. ML methods were −8.28 to −0.23 D, −8.19 to −0.15 D, and −3.38 to 3.51 D, respectively. **Conclusions:** There was a moderate agreement between AA obtained with subjective methods and objective Nidek measurements. The objective AA measurements obtained with a new Nidek autorefractor were significantly lower than subjective measurements.

## 1. Introduction

The accommodative capacity of the eye decreases with age, and this decline becomes clinically relevant from the age of 40 years, resulting in presbyopia [1]. In younger patients, accommodative insufficiency (AI) is a cause of visual fatigue and ocular asthenopia [2], with the prevalence of AI ranging between 1.9% and 14.7% in schoolchildren and young adults [3]. Previous studies have shown that the agreement between subjective methods for measuring the amplitude of accommodation (AA) can show significant variability [4,5]. Thus, objective measurements in which the oculomotor response to an accommodative stimulus is assessed might provide additional value.

Open-field autorefractors, such as Shin-Nippon SRW-5000 (Rexxam Co., Ltd., Osaka, Japan) or Grand-Seiko WR-5100K (Grand Seiko Co., Ltd., Hiroshima, Japan) models, allow measurements to be obtained while presenting accommodative targets [6]. Viewing through an open view is deliberated as a more natural situation that permits a “natural world” environment [7]. Open-field autorefractors have been shown to provide objective AA measurements in several clinical studies [7,8,9,10,11]. Due to their construction, open-field devices allow proximal or lens stimulation to induce an oculomotor response. However, they are mainly used in clinical investigations anddo not have a large market share. In contrast, closed-field autorefractors are typically found in clinical practice, are less expensive, and use internal fixation targets [12]. Although autorefractors have been used for measuring refraction since the 1990s, evaluation of AA in commonly used devices is not available [13]. Nidek Co., Ltd. (Tokyo, Japan) has introduced objective AA assessment into a new series of closed-field autorefractors. This study aims to compare AA measurements obtained using a new-generation autorefractor with conventional subjective methods.

## 2. Materials and Methods

This cross-sectional study enrolled volunteers with healthy eyes at Hygeia Clinic, Gdansk, Poland, in October 2020. The study adhered to the tenets of the Declaration of Helsinki for the use of human participants in biomedical research and was approved by the local ethics committee (KB-21/20). All participants signed informed consent forms after receiving an explanation of the study’s objectives.

Before enrollment, the clinical history was collected and both eyes received a complete ophthalmic examination, including assessment of objective refraction refined by the subjective method with the corrected ametropia most plus (CAMP) lens and by accommodative balancing [14]. The inclusion criteria were an age range of 18 to 50 years; best-corrected visual acuity of at least 0.0 logMAR or better in each eye at 4 m and 40 cm; and no abnormalities of binocular vision at a distance or near, as assessed with a cover test. Subjects with eye diseases, including strabismus, cataract, macular disorders, or inflammation, as well as having undergone intraocular surgery or trauma were excluded. In addition, individuals with a history of systemic diseases, such as diabetes mellitus, anemia, or neurological disorders, and with a history of using ocular and systemic drugs were excluded from this study. The subjective refraction was assessed and subjects were classified as having myopia if the spherical equivalent (SE) was equal to or less than −0.50 D; hyperopia was defined as an SE greater than +1.00 D; and emmetropia, defined as an SE between +1.00 D and −0.25 D [15].

### 2.1. AA Assessment

Measurements were performed by one of the three experienced examiners (K.P., J.S., and P.K.). For each participant, one examiner assessed AA monocularly in both eyes using three methods in random order; all examinations were performed between 3:00 p.m. and 8:00 p.m. All measurements were performed only once for each method and in the same environment luminance.

**Figure 1:**
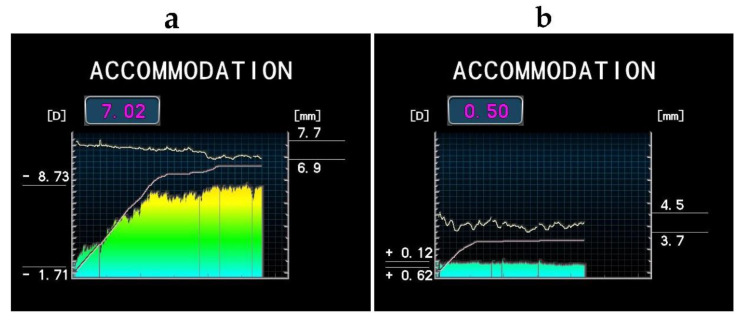
Amplitude of accommodation (AA) and pupil size assessed during the AA measurement in a 25-year-old female (**a**) and a 53-year-old female (**b**). The horizontal axis shows the examination time (up to 30 s). The colored bars represent the real-time refraction, and the lower continuous line represents the internal target position. On the left vertical axis, the minimal and maximal AA values are presented, while the AA magnitude is presented in the box above the chart. The right vertical axis indicates the minimal and maximal pupillary diameter (continuous pupil size assessment is shown as the upper line). All images were obtained with TONOREF IIIL and reproduced with permission from [17].

Automatic AA measurements are implemented in the new series of Nidek infrared autorefractors (ARK-1a, ARK-1s, AR-1a, AR-1s, AR-F, ARK-F, and Tonoref III; Figure 1). During the AA assessment, participants were seated at the instrument (Nidek AR-1a), with their head stabilized in the instrument chin rest and forehead strap. During the measurement, following routine autorefractometry, the participants were requested to focus on a specific fixation target (a picture of a balloon floating over a road). The device uses a Badal optical system, in which a lens moves internally to alter the accommodative stimulus. The measurement was completed after a maximum of 30 s, and the device reduced the measurement time in cases of slow or poor accommodative response. Repeatability and reproducibility of this particular type of Nidek autorefractors in AA measurement [16], as well as the association between the pupil size and AA [17], have already been evaluated; thus, these aspects were not assessed.

Subjective measurements were performed using push-up (PU) and minus-lens (ML) methods. The Royal Air Force (RAF) near-point rule with the reduced Snellen chart was used for the subjective methods. All examinations were performed monocularly under similar testing conditions, with the participants wearing a trial frame with the CAMP lens in front of their eyes. In the PU method, the target was placed 40 cm from the participants. The target was gradually moved closer to the participants at a speed of around 1 cm/s until they reported blur at the 20/30 line; the endpoint was the first sustained blur [18,19]. The distance of the near point of accommodation was read from the scale on the rule to the lens plane in centimeters, converted to diopters, and recorded as AA.

In the ML method, an RAF rule target was placed 33 cm from the participant. With the CAMP lenses in the trial frame, minus lenses were gradually added in 0.50 D steps in front of one eye, while the other eye was occluded. Participants were asked to try to maintain target clarity until the letters became slightly blurred and could not be cleared. Participants were allowed up to 5–10 s for each lens presentation to clear the target. The participants were also offered to take some rest between measurements. AA was determined by the sum of the absolute power of the last lens before reporting slight sustained blur and the dioptric values of the target distance (3 D).

### 2.2. Statistical Analysis

Statistical analysis was performed using MedCalc v. 14 (MedCalc Software bv, Ostend, Belgium) and IBM SPSS Statistics v. 28 (IBM Corporation, Armonk, NY, USA). The normality of data was assessed using the Kolmogorov–Smirnov test, which showed a normal distribution. The results are presented as the mean ± standard deviation (SD). Due to the high correlation in AA measured between the two eyes, only the results for the right eye were analyzed. The dependence between age, examiner, and the results of Nidek, PU, and ML AA was checked using the test of between-subject effects. Comparison between the methods was conducted using repeated-measures analysis of variance (ANOVA) with the Bonferroni post hoc test for multiple comparisons and Pearson correlation coefficients. The Bland and Altman method (plotting the differences between the measurements on the vertical axis against their mean on the horizontal axis) was used to assess the absolute agreement between different techniques [20]. Correlation coefficient values between 0 and 0.3 were considered weak positive; between 0.3 and 0.7, moderate positive; and between 0.7 and 1.0, strong positive linear relationships [21]. In addition, the 95% limits of agreements (LoA; the mean ± 1.96 SD of the differences between the two measurement techniques) were calculated. A *p* value of less than 0.05 was considered significant statistically.

The PS program (version 3.1.6) was used for power and sample size calculations [22]. A sample size of 54 eyes per group was estimated to detect a difference in an AA of 0.5 D, based on a standard deviation of the difference between AA methods of 1.0 D, and a power of 95% at a significance level of 5%.

## 3. Results

**Figure 2:**
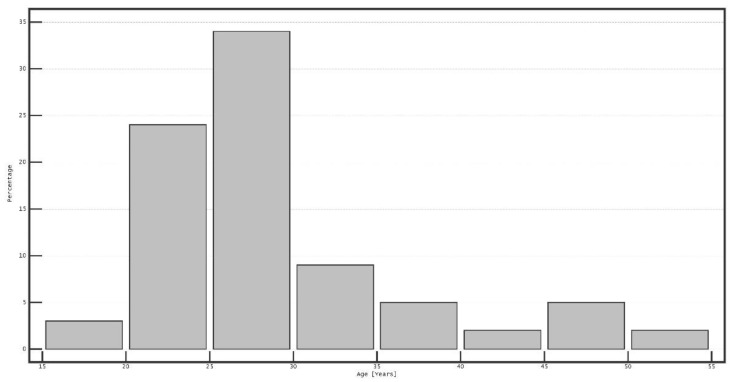
Age distribution of the participants.

Of the 84 subjects who participated in this study, 51 (60.7%) were female. The mean age was 28.6 ± 7.7 years, with a range of 19 to 50 years; the age distribution is presented in Figure 2. The mean spherical equivalent refraction was −0.93 ± 1.73 D (range −7.25 to 2.88 D), while the mean refractive cylinder was −0.62 ± 0.54 D (range −3.25 to 0 D). Thirty-eight eyes were myopic (45.2%), while six eyes (7.1%) were hyperopic; the remaining patients were classified as emmetropes.

The mean ± SD of AA measured using the Nidek autorefractor and the PU and ML techniques was 3.43 ± 1.94 D (95% CI: 3.01 to 3.86), 7.67 ± 2.38 D (95% CI: 7.14 to 8.19), and 7.60 ± 2.81 D (95% CI: 6.98 to 8.21), respectively. There was a statistically significant difference between the different techniques using repeated-measures analysis of variance (*p* < 0.001). AA measured with the Nidek autorefractor and the PU and ML methods was dependent on age (*p* < 0.001, *p* < 0.001, and *p* < 0.001, respectively) but was independent of the examiner performing the evaluation (*p* = 0.434, *p* = 0.166, and *p* = 0.868, respectively). The pairwise comparisons showed that AA obtained with the Nidek autorefractor was significantly lower than that obtained by the PU (*p* < 0.01) and ML (*p* < 0.01) methods, while the difference between PU and ML methods was not statistically significant (*p* = 0.72).

**Figure 3:**
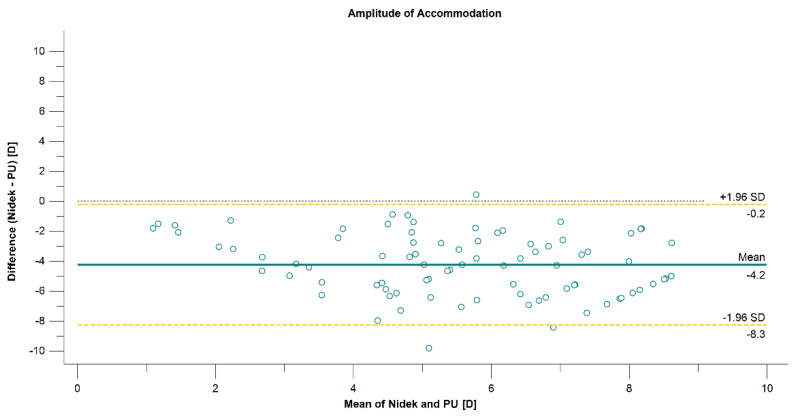
Agreement in amplitude-of-accommodation measurements between the Nidek autorefractor and the push-up (PU) method associated with a 95% CI for difference.

**Figure 4:**
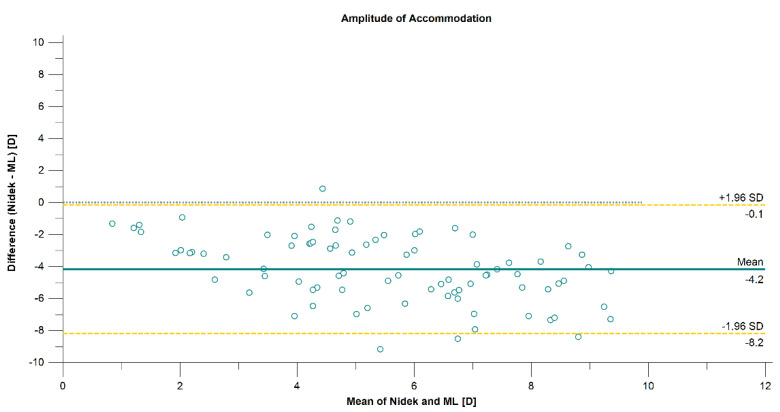
Agreement in amplitude-of-accommodation measurements between the Nidek autorefractor and the minus-lens (ML) method associated with 95% CI for difference.

**Figure 5:**
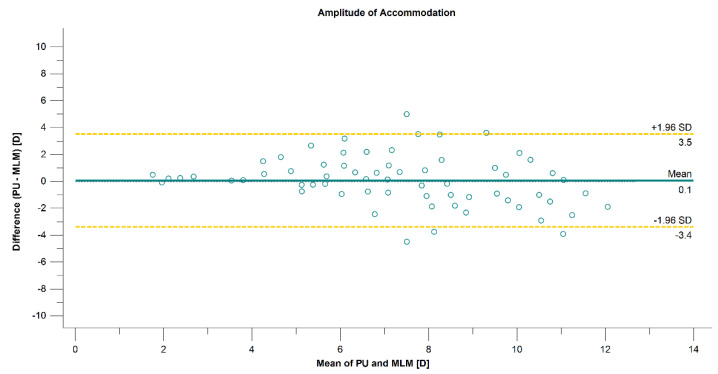
Agreement in amplitude-of-accommodation measurements between the push-up and minus-lens methods associated with 95% CI for difference.

The mean difference, the limit of agreement, and the correlation between the three techniques are presented in Table 1. The correlation was strong between PU and ML (*r* = 0.7821, *p* < 0.001) methods and moderate when comparing Nidek measurements with PU and ML (*r* = 0.5502, *p* < 0.001, and *r* = 0.6832, *p* < 0.001, respectively) methods. The 95% LoA between the methods was over 8.0 D when comparing the Nidek measurements and subjective methods, which are presented on the Bland–Altman plots in Figure 3 and Figure 4. The 95% LoA was slightly lower, approximately 7.0 D, between PU and ML methods (Figure 5).

**Figure 6:**
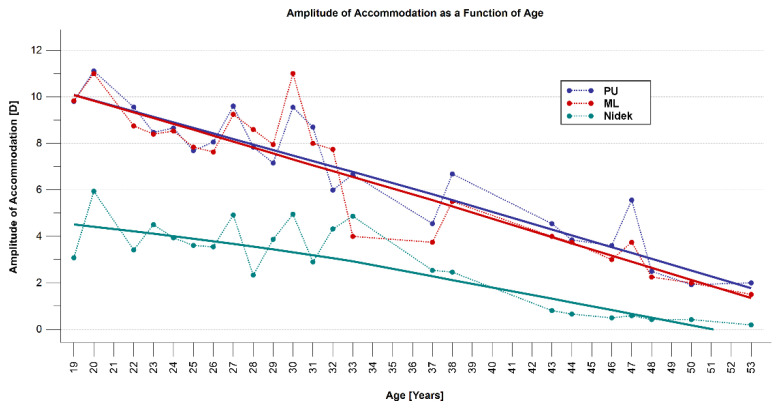
Scatter plot showing the amplitude of accommodation (AA) obtained with all the three methods: subjective push-up (PU), subjective minus-lens (ML), and objective Nidek methods as a function of age.

AA as a function of age is illustrated in Figure 6. AA gradually decreased in all three methods; the slope of the decrease was greater in subjective methods. The decrease in AA was more prominent after 31 years of age using objective AA.

## 4. Discussion

Subjective measurements overestimate AA compared to objective methods. For example, Ostrin and Glasser reported a difference of up to four diopters in AA measured using subjective techniques compared to objective methods in presbyopic subjects [23]. At approximately 50 years of age, AA evaluated in objective measurements effectively becomes zero [24,25], while the apparent residual 1.00 D accommodation using PU and ML methods really reflects the eye’s depth of focus. In optics, the depth of focus is the distance through which the test target can be displaced without initiation of the perception of blur; the smaller the aperture, the wider the depth of field. Thus, greater retinal illumination or pupil constriction without an increase in illumination, e.g., due to an accommodative stimulus, would increase the depth of field. It was hypothesized that subjective AA assessment is an inadequate measure to evaluate true accommodation, because it fails to differentiate between passive depth of focus and an active accommodative power change in the eye [26,27]. Some other reasons for this overestimation include relative distance magnification or stimulation of proximal accommodation in the PU method, the influence of the depth of focus, differences in retinal illumination and pupillary size during measurement, and inaccuracies in measuring the distance from the near point of accommodation to the spectacle plane (particularly at shorter distances, where small differences in metric units result in large differences in dioptric equivalents) [28]. Objective techniques might quantify the magnitude of refractive change of the eye to more accurately and precisely depict accommodative ability [23,29]. Particularly in younger age groups, the mean objective amplitudes are approximately half of those measured with the subjective PU test in the same subjects [27].

Moreover, objective AA measurements are known to be greater with the proximal stimulated technique than with lens stimulation [27,30]. Table 2 summarizes studies comparing objective AA measurements taken with subjective methods. The specific construction of the Nidek device makes it possible to trigger the accommodative response by only lens stimulation. As this is a closed-field autorefractor, the monocular depth cues are absent, the target size and luminance do not change with the stimulus, and there are no familiarly sized objects and no interposition of targets in depth [31,32]. This could explain why the results are even lower than those with lens-stimulated AA in open-field autorefractors.

Potential advantages of automatic objective measurement is the ease of performance, as it only requires one to focus the camera on the center of the pupil and press the trigger; the measurement can be conducted by a less skilled examiner as it does not require evaluation of subjective refraction. Moreover, it excellently fits into the work streaming process. The time required to obtain an objective measurement with the Nidek device might be similar to the subjective assessment by an experienced examiner. The duration of Nidek AA measurement for each eye is up to 30 s; however, it is shorter if no response to the accommodative stimulus is obtained for a few seconds. However, to be able to perform a scan, the patient must be able to fixate sufficiently for up to 30 s during the measuring process; potentially, this could be problematic when evaluating children, elder patients, and patients with nystagmus.

Assessing AA as a function of age shows that it progressively decreases from around 5 years of age to around 50 years. Clinicians are sometimes surprised that accommodation ceases as soon as the early fifties [25]; the data reported in this study support this view. The variation in accommodative amplitude with age showed that objective AA drops rapidly after about the age of 38 years, which is almost coincident with the age of onset of presbyopia, while changes in subjective AA techniques are associated with a gradual decrease even up to the age of 50 years. Age-related changes in objective AA in this study were somewhat curvilinear and did not follow the sinusoidal function with a rapid decrease in accommodation in the age range of 20 to 50 years reported by Anderson et al. [33].

Previous studies have also shown that AA measurements show higher values in the PU method than in the ML technique [4,34]. In our study, measurements were taken in a random order and the difference between the AA assessed with PU and ML methods was less than 0.1 D. A variety of factors might influence subjective AA assessment, including variations in RAF ruler manufacture, specification of the measurement endpoint, specification of the reference point of measurement, measurement conditions, consideration of refractive error, and psychological factors [35,36]. Atchison et al. recommended using letter discrimination rather than blur perception for the PU method, and the latter method, which was used in our study, could lead to underestimation of AA [37]. Moreover, it is known that for young subjects with high amplitudes, it is difficult to get an accurate measurement in the PU method, as small distance changes correspond to large dioptric values [37].

One could consider that it would be more proper to compare objective findings from the new Nidek device to objective amplitudes obtained with the Grand Seiko open-field autorefractor method mentioned in previous publications. Nevertheless, this study aimed to assess the clinical utility of the new autorefractor by comparing it with the currently used methods. These results could be particularly useful when comparing AA values obtained with conventional subjective methods and the results of Nidek devices.

In conclusion, the objective AA measurements obtained with the new Nidek autorefractor were significantly lower than subjective measurements and showed moderate agreement.

## Figures and Tables

**Table 1:** Mean differences, 95% limits of agreement (LoA), correlation coefficient (*r*), and pairwise comparison between various methods for assessing the amplitude of accommodation (AA); (*n* = 84 eyes).

Pairwise Comparison	Mean Difference ± SD (95% CI) [D]	95% LoA [D]	*r* (*p* Value)	*p* Value
Nidek-PU	−4.23 ± 2.04 (−4.79 to −3.68)	−8.28 to −0.23	0.5502 (<0.001)	<0.001
Nidek-ML	−4.16 ± 2.03 (–4.71 to −3.61)	−8.19 to −0.15	0.6832 (<0.001)	<0.001
PU-ML	0.07 ± 1.75 (−0.40 to 0.55)	−3.38 to 3.51	0.7821 (<0.001)	1.0

CI: confidence interval; ML: minus lens; PU: push-up; SD: standard deviation.

**Table 2:** Studies comparing the objective amplitude of accommodation obtained with commercially available autorefractors inducing 5 D accommodative stimulation using lens or proximity stimulus with subjective measurements.

Study	Age (Years)	Number of Eyes	Device	Sub. PU AA (D)	Obj. Proximal-Stimulated AA (D)	Obj. Lens-Stimulated AA (D)
Anderson and Stuebing [27]	26–30	25	Grand Seiko WAM-5500	8.45 ± 2.24	6.05 ± 1.1	5.7 ± 1.1
Win-Hall et al. 2007 [30]	21–30	22	Grand Seiko WR-5100K	7.74 ± 0.36	4.68 ± 0.10 *	4.13 ± 0.09 *
Present study	19–50	84	Nidek AR-1a	7.67 ± 2.38	N/A	3.43 ± 1.94

* 5 D accommodative stimulation using lens or proximity stimulus; AA: amplitude of accommodation; PU: push-up; Sub: subjective; Obj: objective.

## Data Availability

Not applicable.

## Abbreviations

AA: amplitude of accommodation; AI: accommodative insufficiency; CI: confidence interval; ML: minus lens; PU: push-up; RAF: Royal Air Force; SD: standard deviation.
